# MMP-14 Exhibits Greater Expression, Content and Activity Compared to MMP-15 in Human Renal Carcinoma

**DOI:** 10.3390/ijms25158107

**Published:** 2024-07-25

**Authors:** Grzegorz Młynarczyk, Anna Tokarzewicz, Monika Gudowska-Sawczuk, Barbara Mroczko, Vojtěch Novák, Adam Novák, Przemysław Mitura, Lech Romanowicz

**Affiliations:** 1 Department of Urology, Medical University of Białystok, 15-276 Białystok, Poland; 2 Department of Medical Biochemistry, Medical University of Białystok, ul. Mickiewicza 2, 15-089 Białystok, Poland; anna.tokarzewicz@umb.edu.pl (A.T.); lech.romanowicz@umb.edu.pl (L.R.); 3 Department of Biochemical Diagnostics, Medical University of Białystok, 15-269 Białystok, Poland; monika.gudowska-sawczuk@umb.edu.pl (M.G.-S.); barbara.mroczko@umb.edu.pl (B.M.); 4 Department of Neurodegeneration Diagnostics, Medical University of Bialystok, 15-269 Białystok, Poland; 5 Department of Urology, Charles University and University Hospital Motol, 150 06 Praha, Czech Republic; vojtech.novak.lf2@gmail.com (V.N.); adamnovak1229@gmail.com (A.N.); 6 Department of Urology and Oncological Urology, Medical University of Lublin, 20-059 Lublin, Poland; przemyslawmitura@umlub.pl

**Keywords:** cancer, renal carcinoma, transmembrane metalloproteinases, MMP-14, MMP-15

## Abstract

Membrane-type metalloproteinases (including MMP-14 and MMP-15) are enzymes involved in the degradation of extracellular matrix components. In cancer, they are involved in processes such as cellular invasion, angiogenesis and metastasis. Therefore, the aim of this study was to evaluate the expression, content and activity of MMP-14 and MMP-15 in human renal cell carcinoma. Samples of healthy kidney tissue (n = 20) and tissue from clear-cell kidney cancer (n = 20) were examined. The presence and contents of the MMPs were assessed using Western blot and ELISA techniques, respectively. Their activity—both actual and specific—was evaluated using fluorimetric analysis. Both control and cancer human kidney tissues contain MMP-14 and MMP-15 enzymes in the form of high-molecular-weight complexes. Moreover, these enzymes occur in both active and latent forms. Their content in cancer tissues is very similar, but with a noteworthy decrease in content with an increase in the kidney cancer grade for both membrane-type metalloproteinases. Even more notable is the highest content of the investigated enzymes represented by MMP-14 in the control tissues. Considering the actual and specific activity outcomes, MMP-14 dominates over MMP-15 in all of the investigated tissues. Nevertheless, we also noted a significant enhancement of the activity of both metalloproteinases with an increase in the grade of renal cancer. The expression and activity of both enzymes were detected in all examined renal cancer tissues. However, our findings suggest that transmembrane metalloproteinase 14 (MMP-14) plays a much more significant and essential role than MMP-15 in the studied renal carcinoma tissues. Therefore, it seems that MMP-14 could be a promising target in the diagnosis, prognosis and therapy of renal cell carcinoma.

## 1. Introduction

The kidneys are even, parenchymal organs located in the retroperitoneal space of the human abdominal cavity. Their main functions are the excretion of metabolic products and the regulation of the components, volume and pH of body fluids. They also have metabolic and endocrine functions [1,2,3]. The structure of the extracellular matrix has a significant impact on their proper functioning. Its components, including collagen (the main extracellular protein), glycoproteins and elastin molecules, form a complex network that also interacts with surrounding cells.

The ECM is present in the renal cortex in anatomically distinct areas and has varied functions, depending on its molecular components: glomeruli, tubulointerstitium and in vessels. Besides ECM remodeling, MMPs take part in several other processes, such as basement membrane destruction, angiogenesis, cell migration and cell apoptosis [4,5,6]. The group of membrane-type metalloproteinases consists of six enzymes: MMP-14, MMP-15, MMP-16, MMP-24, MMP-17 and MMP-25. Various components of the extracellular matrix, such as collagen types I, II and III; aggrecan; elastin; fibronectin; gelatin; laminin and MMP-2 and -13, are degraded by these enzymes. At the same time, they are simultaneously involved in signal transfer at the cellular level [7,8,9].

MMP-14, the first discovered membrane-type metalloproteinase, became the most frequently selected subject of MMP research. The main substrates degraded by MMP-14 are type I collagen and pro-MMP-2. Collagen II and III can also be degraded by this enzyme, but to a lesser extent [10]. The enzyme also degrades gelatin and the hyaluronate receptor [11]. To activate MMP-2, MMP-14 forms a ternary complex on the cell surface with TIMP-2 (Tissue Inhibitor of Metalloproteinases 2) and with MMP-2 [12,13,14]. It plays a very important role in angiogenesis. Furthermore, MMP-14 promotes cellular invasion in cancerogenic processes, metastasis, angiogenesis, wound healing, atherosclerosis and rheumatoid arthritis [15,16]. The overexpression of MMP-14 has been observed in kidney fibrosis [17]. Moreover, MMP-14 expression varies with the tumor type and is high in melanomas, mesenchymal tumors and brain tumors [18], as well as in liver tumors and breast cancer [19,20]. Some authors have suggested that MMP-14 can be used to predict cancer prognosis [21].

It has been shown that the active form of MMP-15 plays a role in breast, prostate and colon cancerogenic processes. It degrades only type 1 collagen; however, its specific activity outcomes showed only 1/100 that of MMP-14 activity. Moreover, it was demonstrated that MMP-15 may promote cell invasion into basement membrane matrices [22,23,24].

Transmembrane metalloproteinases MMP-14, MMP-15 and MMP-16 have been found to be significantly expressed in tumor-transformed tissue in renal cell carcinoma and to be present in urine samples from the same patients [25]; however, no reports have been published on their expression in renal cell carcinoma.

Transmembrane metalloproteinases play an important part in renal cancer; however, their significance is not entirely clear. Therefore, the expression, content and activity of MMP-14 and MMP-15 in cancerous tissue in relation to healthy human kidney tissue were investigated.

## 2. Results

### 2.1. MMP-14 and MMP-15 Content in Tissue Samples

Based on the measurements described in Section 4.3, considerable differences in the content (mg/kg of total protein content) of both investigated MMPs in control and renal cell carcinoma samples were noted.

MMP-14 was present in an extremely high quantity in control tissue samples, at approximately 1.1 g of MMP-14/kg of total protein content, regardless of the source of control material (control G2 or control G3). The G2 and G3 tumor samples contained substantially lower amounts of MMP-14. The G2 tumor samples contained 14.87, whilst the control contained 1057 mg of MMP-14/kg of total protein content. Even lower quantities were found in the G3 tumor, as compared to the control tissue (12.93 vs. 1119.8 mg of MMP-14/kg of total protein content). Thus, the progress in tumor invasiveness from tumor grade G2 to G3 involved a rather small decrease in the MMP-14.

**Figure 1:**
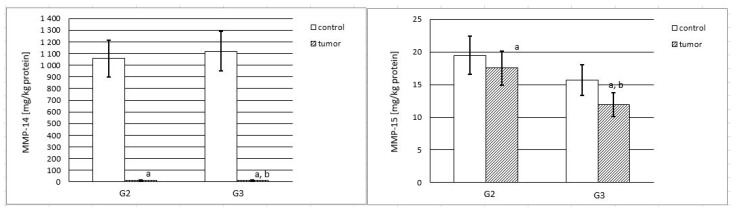
Content of MMP-14 and MMP-15 in control samples and renal cell carcinoma (G2 and G3) samples. MMP-14: a—*p* < 0.001 control vs. renal cell carcinoma; b—*p* < 0.05 G2 vs. G3; MMP-15: a—*p* < 0.05 control vs. renal cell carcinoma; b—*p* < 0.001 G2 vs. G3.

The approximate content of MMP-15 in control samples was 19.48 (control of G2) and 15.69 (control of G3) mg of MMP-15/kg of total protein content. The G2 and G3 tumor samples contained smaller amounts of MMP-15. There was almost 2 mg less of the enzyme in the G2 tumor. An even lower value (approximately 4 mg less of the enzyme) was found in the G3 tumor, compared to the respective control tissue samples. Such progress in tumor invasiveness from tumor grade G2 to G3 involved a significant decrease in the MMP-15 content. The MMP content results are provided in Figure 1.

### 2.2. Western Blot Analysis of MMP-14 and MMP-15

#### 2.2.1. Expression of MMP-14

To determine the expression of MMP-14 in renal cell carcinoma samples and the control samples, Western Blot analysis was performed under both non-reducing and reducing condition. Non-reducing conditions allow the separation of native, free forms of proteins as well as their complexes. The use of a reducing agent breaks the disulphide bonds present in proteins and complexes. It shows the presence of subunits or protein fragments, if they are linked by disulphide bridges. It is also possible that the separation of protein subunits results in the disappearance of protein binding sites that allowed the formation of high-molecular-weight complexes. This causes changes in the Western blot obtained results.

**Figure 2:**
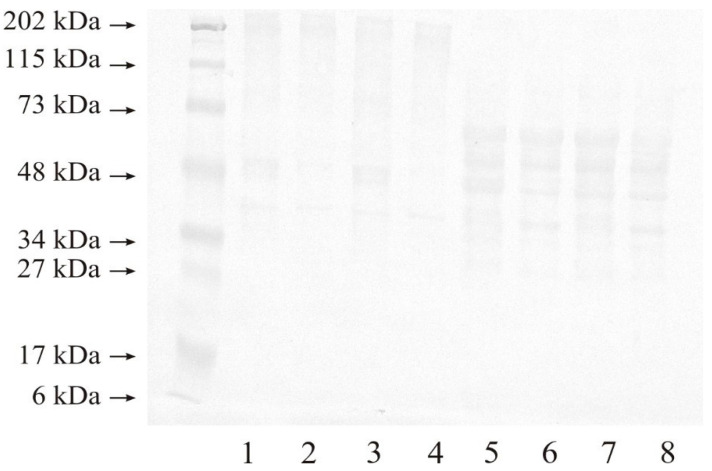
Representative results of Western immunoblot analysis of MMP-14 in renal cell carcinoma. Non-reducing conditions: lane: 1—control sample for grade G2 tumor; 2—grade G2 tumor; 3—control sample for grade G3 tumor; 4—grade G3 tumor. Reducing conditions: lane: 5—control sample for grade G2 tumor; 6—grade G2 tumor; 7—control sample for grade G3 tumor; 8—grade G3 tumor; 20 µg of protein was applied to the gel.

Figure 2 presents the results of a representative analysis, from which it can be seen that all of the tested samples contained MMP-14 in the form of high-molecular-weight complexes, as indicated by an intense band of approximately 202 kDa under non-reducing conditions (lanes 1–4). In addition, both control samples (lane 1: G2 control, and lane 3: G3 control) showed an intense band of approximately 48 kDa in molecular weight, while both tumor samples (lane 2: G2, and lane 4: G3) showed a non-intensive band of the discussed mass. A band of approximately 40 kDa was present in both control samples and renal cell carcinoma samples. No other bands of lower masses were found under non-reducing conditions.

Reduction of the disulphide bridge with 2-mercaptoethanol resulted in the disappearance of high-molecular-weight complexes in all of the tested samples (lanes 5–8). Both control and renal cell carcinoma samples showed bands with approximately 60, 55, 48, and 40 kDa molecular weight. All four samples also demonstrated a non-intensive band with a molecular weight of 30 kDa (lanes 5–8; Figure 2).

#### 2.2.2. Expression of MMP-15

**Figure 3:**
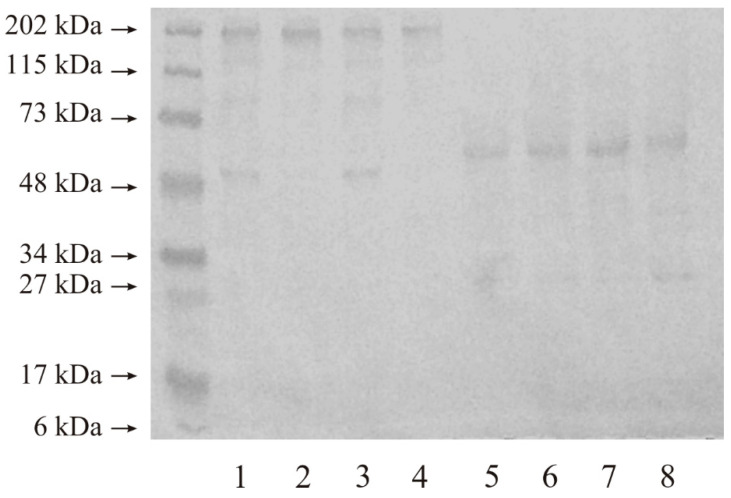
Representative results of Western immunoblot analysis of MMP-15 in renal cell carcinoma. Non-reducing conditions: lane: 1—control sample for grade G2 tumor; 2—grade G2 tumor; 3—control sample for grade G3 tumor; 4—grade G3 tumor. Reducing conditions: lane: 5—control sample for grade G2 tumor; 6—grade G2 tumor; 7—control sample for grade G3 tumor; 8—grade G3 tumor; 20 µg of protein was applied to the gel.

Figure 3 shows that applying even a low mass of protein (20 micrograms) to the gel resulted in the appearance of intense bands with molecular masses of approximately 202 kDa and 48 kDa in all of the tested samples, without any other bands of lower masses, under non-reducing conditions (lanes 1–4). After the reduction of disulphide bonds, only two bands (approximately 50 kDa and 30 kDa) can be seen on the Western Blot gel, which was separated and present in all the examined tissues (lanes 5–8).

### 2.3. Activities of MMP-14 and MMP-15

The actual and specific activities of both MMPs were evaluated through fluorimetric assay with an oligopeptide as a substrate. Each of the discussed enzymes was isolated on a microplate, which was pre-coated with a suitable and selective antibody. After immobilization of the enzymes, all remaining proteins were washed out from the microplate. The quantity of enzyme degradation product was determined after the addition of fluorogenic substrate.

The actual activity of membrane-type matrix metalloproteinases allows for the comparison of the activity of all the enzymes tested with each other (with the total protein content of the tissue extract as a common denominator). Moreover, the specific activity allowed for determination of what part of the enzyme was in the active form, without an inhibitor bound to the enzyme’s active site.

#### 2.3.1. Actual and Specific Activity of MMP-14

**Figure 4:**
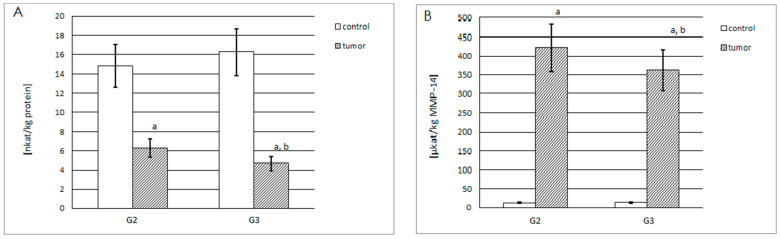
(**A**) Actual activity of MMP-14 (nkatal/kg of total protein content) in control samples (total n = 20), and grade G2 (n = 10) and G3 (n = 10) renal cell carcinoma samples; a, *p* < 0.001 cancer vs. respective control sample; b, *p* < 0.001 grade G3 vs. G2 renal cell carcinoma samples. (**B**) Specific activity of MMP-14 (µkatals/kg of the MMP-14) in the same samples (each group n = 10); a, *p* < 0.001 cancer vs. respective control sample; b, *p* < 0.05 grade G3 vs. G2 renal cell carcinoma samples.

As shown in Figure 4A, similar actual activity of MMP-14 (approximately 15 nkatal/kg of protein content) was found in all control samples. The determined actual activity was more than two times lower in G2 tumor samples and almost four times lower in G3 tumor samples when compared to corresponding control samples; therefore, the actual activity significantly decreased with increasing grade of renal cell carcinoma.

In all of the tested samples, the content of MMP-14 was measured beforehand, which enabled the possibility of determining the specific activity of MMP-14 (µkatal/kg of MMP-14). From Figure 4B, it can be seen that both control samples were characterized by very low and similar specific activity, approximately 14 µkatals/kg of MMP-14. In addition, specific activity in the G2 and G3 tumor samples was nearly 30 times and more than 25 times higher in comparison to the control samples, respectively.

#### 2.3.2. Actual and Specific Activity of MMP-15

**Figure 5:**
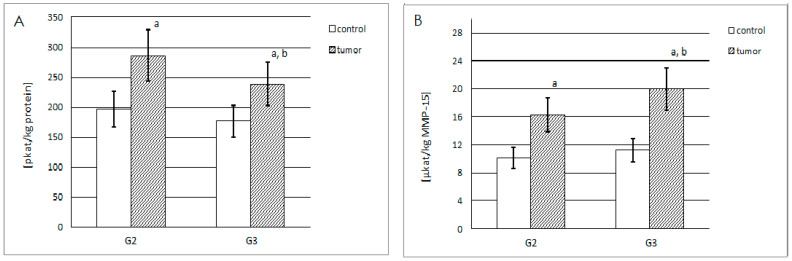
(**A**) Actual activity of the MMP-15 (pkatals/kg of total protein content) in control samples (total n = 20), and grade G2 (n = 10) and G3 (n = 10) renal cell carcinoma samples; a, *p* < 0.001 cancer vs. respective control samples; b, *p* < 0.05 grade G3 vs. G2 renal cell carcinoma samples. (**B**) Specific activity of the MMP-15 (µkatals/kg of the MMP-15) in the same samples (each group n = 10); a, *p* < 0.001 cancer vs. respective control samples; b, *p* < 0.05 grade G3 vs. G2 renal cell carcinoma samples.

The highest actual MMP-15 activity (pkatals/kg of total protein content) was found in the grade G2 renal cell carcinoma samples (Figure 5A). The increase in the grade of renal cell carcinoma was accompanied by a decrease in enzyme activity; however, it was still significantly higher than in the corresponding control samples. The actual activity was 35% higher in G3 and 45% higher in G2 tumor samples, compared to the corresponding control samples.

The specific activity (µkatals/kg of MMP-15) of this enzyme was also determined. Figure 5B shows that this type of activity was more than 1.5 times higher in G2 tumor samples, compared with the respective control samples. The G3 tumor samples demonstrated the highest specific activity of MMP-15—approximately 1.8 times higher than in the respective control sample and over 22% higher than in the G2 tumor samples.

## 3. Discussion

A small amount of extracellular matrix has been observed in the human kidney. In addition to collagen, the ECM contains many other important components, such as elastin, proteoglycans and structural glycoproteins. The basement membrane, which separates epithelial tissue from connective tissue, is a special layer of the ECM. This membrane is mainly composed of type IV collagen and glycoproteins [26,27,28]. Many researchers have investigated the characteristics of human renal cell carcinoma [29,30,31,32]. The focus of the study described in this article was to evaluate the expression, content and activity of the MMPs involved in the degradation of ECM components in human kidney tissues.

Membrane-type metalloproteinases are represented by six enzymes. We compared MMP-14 (membrane-type MMP 1) and MMP-15 (membrane-type MMP 2) in human kidney tumor to those in healthy parts of the same organ, which were used as a control material as ethical concerns excluded the possibility of taking a kidney sample from healthy donors. Post-mortem human kidney collection entails a substantial change in protein content and is possible after several hours. After such time, the tissue degradation process is usually advanced. According to Lee et al. [33], all of the enzymes degrading the extracellular matrix are only active components. The content of protein is much lower, when compared with the levels found in tissue that has been newly taken from a living organism.

ELISA was used to measure the total content of the tested MMP-14 and MMP-15 in kidney samples. We observed that human control kidney tissue showed higher amounts of both MMPs, compared to tumor tissue. Based on this, it can be speculated that both enzymes may play an important role in the restoration of the extracellular matrix in healthy kidneys. It is worth noting that the highest content was demonstrated in both control tissues for MMP-14, while a very similar amount of both enzymes was found in both grades of cancer. The above outcomes demonstrate that the synthesis and secretion of investigated metalloproteinases from cells were not inhibited. Moreover, there are differences between malignant and normal renal cells in terms of enzyme secretion outside the cell. The synthesis of both transmembrane metalloproteinases may be at a similar level for both kinds of cells, but their secretion to the extracellular space is limited by cancer cells. It is common knowledge that protein turnover is much faster inside the cell than outside it [34]. MMPs, similar to all other matrix metalloproteinases, are extracellular enzymes and, therefore, their lower content in the tumor tissue may be explained by the decrease in enzyme secretion from malignant cells.

On the basis of the actual activity of the tested MMPs (katal per kg of total protein content), the activity of the two analyzed MMPs was compared in different tissue samples. Based on the results, it can be concluded that MMP-14 is significantly less active in tumor tissue than in control tissue; at the same time, it is also approximately 70 times more active in healthy human kidney samples than MMP-15. Based on this, it seems that MMP-14 is significantly involved in maintaining ECM homeostasis in healthy kidneys.

MMP-15 demonstrated a significantly higher activity in both cancer grades, as compared to the control material. However, MMP-14 demonstrated a much higher activity in both grades of kidney cancer, when compared to MMP-15. Such a difference in the actual activity of both investigated enzymes may provide further proof explaining the significant role of MMP-14 in carcinogenic processes, as well as indicating that it does not reduce the role of MMP-15.

Furthermore, the activities of MMP-14 and MMP-15 in the cells of both tumor grades were determined. However, there was no relation between enzyme expression/activity and cancer progression.

Based on the specific activity results for each of the MMPs tested (µkatal per kg of enzyme), it can be observed that part of the enzyme tested was present in the active form, without an inhibitor bound to the active site of the enzyme. Both examined enzymes showed a similar specific activity in the control tissue. Furthermore, both enzymes demonstrated a significantly higher activity in the cancerous material compared to the control tissue. In addition, a significantly higher specific activity of MMP-14 in tumor tissue was determined, compared to MMP-15. This appears to be related to the wide range of proteins degraded by MMP-14, including pro-MMP-2 activation [12,13,14]. In summary, it can be concluded that most MMP-14, unlike MMP-15, remains in an active form without the inhibitory effect of TIMPs. It seems that the MMP-14 enzyme showed greater enzymatic activity compared to MMP-15. Other authors have also demonstrated the over-expression of MMP-14 at its mRNA stage in various types of tumors [18,19,20].

As shown by the Western blot analysis results, both studied enzymes were present in kidney tissues primarily in the form of high-molecular-weight complexes. This is due to the fact that the extracellular matrix is a tissue structure in which different proteins interact with each other. In many cases, these interactions do not affect enzyme activity. The described complexes disappear as a result of the reduction of disulphide bridges. This means that the bonds connecting all components are weak and non-covalent.

In all tested tissue samples, MMP-14 was present in a free active form. This is evidenced by the presence of a band with a molecular weight of approximately 50 kDa, both before and after disulphide bond reduction. The obtained results also demonstrated the presence of MMP-15 in free active form in all samples, due to a clearly visible band with a molecular weight of 50 kDa, both before and after the reduction of disulphide bonds.

The results of other studies have demonstrated that the transmembrane metalloproteinases examined in this study were significantly expressed during the course of renal cell carcinoma in tumor-lesioned tissue and were present in urine samples from the same patients [25]. However, published reports on the expression of these enzymes in renal cell carcinoma are still lacking.

Even though the content of both investigated membrane-type metalloproteinases in renal cancer was similar, the activity of MMP-14 was much higher in both tumor phases compared to that of MMP-15. This may mean that the catalytic ability of MMP-14 is much higher in comparison to MMP-15. Despite their similar contents, MMP-14 seems to be the dominant MMP in kidney cancer. On the other hand, the differences between outcomes for both enzymes may point to their specific involvement in a particular growth period and in cancer differentiation. Furthermore, the above outcomes show that cancerous cells of grade G2 and G3 kidney tumors may enhance the activity of transmembrane metalloproteinases. Based on the abovementioned similar content of both enzymes and the lower specific activity of MMP-15 in comparison with MMP-14, it can be stated that many more molecules of MMP-15 in an inactive form were present. Such differences in the activity of both MMPs proved that they participated in the opposite way in different phases of ECM remodeling, and at different stages of tumor development. This indicates differences in regulating the expression and activation of the examined matrix metalloproteinases in human kidney tumors. Due to the fact that the control sample was taken from the same kidney, it is possible that there was a carcinogenic influence on the metabolism of the entire organ. It may be assumed that the initiation of ECM component degradation in the extracellular matrix is the most important stage in the process of tumor growth. The tissue material was used, instead of urine or blood serum of the same patient, in order to better reflect the roles of membrane-type metalloproteinases in tissue metabolism.

## 4. Materials and Methods

The study protocol was approved by the Bioethical Committee of the Medical University of Bialystok.

### 4.1. Tissue Material

The sample was composed of patients with clear-cell kidney cancer of grade G2 or G3 according to the ISUP/WHO malignancy scale. All patients underwent radical surgical treatment, namely open nephrectomy. A total of 20 patients (6 females and 14 males) aged between 48 and 78 years (mean age, 61 years) were selected for this study. The sample material included specimens of primary tumors collected during the surgery. Patients with cancers of grades G2 (n = 10) and G3 (n = 10) were selected as participants for this study. Tissue specimens of the same kidney, but always located opposite to the tumor, were collected as a comparative material. Any carcinogenetic lesion was macroscopically visible in the control tissue. All samples taken were washed using 0.9% NaCl solution, weighed, portioned and stored at −70 °C during the entire period of analysis. The surgical procedures were performed at the Department of Urology of the University Hospital in Bialystok.

### 4.2. Specimen Preparation

A 0.9% NaCl solution was used to wash the tissue samples. The tissues were then cut into small pieces, which were used to prepare the homogenate. Tris/HCl 0.05 M buffer (pH = 7.4) was used to prepare tissue homogenates (20%, *w*/*v*). A knife homogenizer (20,000 rpm, 3 × 30 s, 0 °C) was used for homogenization. In addition, homogenates were subjected to ultrasonification (20 kHz, 3 × 15 s, 0 °C). Then, centrifugation was performed at 10,000× *g* for 30 min at 4 °C. The supernatants (final tissue samples) obtained after centrifugation were collected, divided, and stored at −70 °C for further use.

### 4.3. MMP Contents

An MMP-14 ELISA kit provided by Sigma-Aldrich (Saint Louis, MO, USA; cat. no. RAB0362) and MMP-15 ELISA kit provided by Cloud-Clone Corp. (Katy, TX, USA; cat. no. SEA099Hu) were used to determine the contents of MMP-14 and MMP-15, respectively, in the test samples. Both quantitative assays were performed according to the manufacturer’s instructions.

### 4.4. MMP Western Blot

Laemmli’s method [35] was used for electrophoresis of the tested tissue samples (20 μg of protein for MMP-14 self and MMP-15). It was carried out using a 10% SDS-polyacrylamide gel under non-reducing and reducing conditions. The gels were then blotted for 1 h on nitrocellulose membranes (Sigma) at 100 mA. To block the membranes, they were soaked in 5% (*w*/*v*) skimmed milk powder in TBS-T solution (20 mM Tris/HCl buffer, pH 7.4, 150 mM NaCl, 0.05% (*v*/*v*) Tween 20) for 1 h. After this step, the membranes were incubated with a monoclonal antibody directed against human MMP-14 (R&D systems, Minneapolis, MN, USA; cat. no. MAB918) or a monoclonal antibody directed against human MMP-15 (R&D systems, Minneapolis, MN, USA; cat. no. MAB916) in TBS-T containing 1% bovine serum albumin (*w*/*v*), overnight at 4 °C. Membranes were then washed several times in TBS-T buffer. Bound antibodies were detected using secondary antibodies conjugated to alkaline phosphatase. For this, the membranes were placed in a solution of secondary antibody for 1 h at room temperature with gentle mixing. After this step, BCIP/NBT reagent (Sigma-Aldrich (Saint Louis, MO, USA; cat. no. B1911) was added. Pre-stained molecular weight markers (BioRad, Hercules, CA, USA) were used to estimate the molecular weight of MMPs. Representative blots that were obtained are shown in the manuscript.

### 4.5. Actual and Specific Activities of MMPs

The actual and specific activities of MMP-14 and MMP-15 were measured using a black 96-well flat-bottom microplate that was pre-coated with the appropriate MMP-specific antibody; in particular, the same antibodies previously used in the Western blot analysis were used to coat the wells [34].

The immobilization of the metalloproteinase was achieved by adding 100 microliters of respective sample to each well. Next, the microplate with samples was incubated overnight at 4 °C. TBS-T Buffer (50 mM Tris/HCl pH 7.4, 0.9% NaCl, 0.05% Tween 20) was used to wash out any remaining proteins not bound to the microplate. The activity of the tested MMPs was determined in 100 µL of 50 mM Tris/HCl buffer at pH 7.5 (10 mM CaCl_2_, 150 mM NaCl, and 0.025% Brij 35) [9] and using MCA-Pro-Leu-Ala-Cys(p-OMeBz)-Trp-Ala-Arg(Dpa)-H2 (Merck, Darmstadt, Germany; cat. no. 444258) as a fluorogenic substrate (4 μM final concentration). For this purpose, the microplate was incubated for 60 min at 37 °C with gentle shaking. After incubation, the reaction was stopped by adding 25 μL of 100 mM EDTANa_2_. Using a spectrophotofluorimetric/multimode microplate reader (Tecan Infinite® 200 PRO, Männedorf, Switzerland; excitation wavelength 325 nm, emission wavelength 393 nm), the degradation of the fluorogenic substrate was determined. Then, using a calibration curve prepared under the same conditions with 7-amino-4-methylcoumarin (Sigma-Aldrich, Saint Louis, MO, USA; cat. no. 257370) as a standard, the amount of degraded substrate was measured.

The actual activity of each tested MMP was calculated as enzyme activity per kg of total protein content (catal/kg of total protein content). The specific activity, on the other hand, was calculated as the enzyme activity per kg of MMP content in the sample (catal/kg of MMP content).

### 4.6. Protein Determination

The Bradford protein assay was used to measure the protein concentration [36].

### 4.7. Statistical Analysis

Mean values were calculated based on 10 obtained results for each tested group. Their standard deviations (SD) were also determined. Statistical analysis was carried out using Student’s *t*-test, taking *p* < 0.05 as indicating statistical significance.

## 5. Conclusions

Urinary tract cancers constitute an important diagnostic and therapeutic problem, and their incidence increases every year. Renal cell carcinoma is the most common type of malignant tumor in the kidney. Extracellular matrix metalloproteinases play an important role in cancer processes due to their involvement in local tumor invasion, the formation of distant metastases, and the process of angiogenesis. The aim of this study was to evaluate the expression, content, and activity of selected membrane-type metalloproteinases (MMP-14 and MMP-15) in human renal cell carcinoma. The research and analysis carried out showed that both control and cancerous human kidney tissues contain MMP-14 and MMP-15 enzymes in high-molecular complexes, in both active and latent forms. Their content is similar in cancer tissues but decreases with higher kidney cancer grades. Notably, MMP-14 is most abundant in control tissues and dominates over MMP-15 in all tissues. The activity of both enzymes increases with cancer grade. MMP-14 appears to play a more significant role than MMP-15 in renal carcinoma, suggesting it could be a promising target for diagnosis, prognosis, and therapy in renal cell carcinoma.

## Data Availability

All data generated or analyzed during this study are included in this article. Further enquiries can be directed to the corresponding author.
